# The biosafety incident response competence scale for clinical nursing staff: a development and validation study

**DOI:** 10.1186/s12912-024-01848-6

**Published:** 2024-03-14

**Authors:** Chao Wu, Hongli Zhang, Yinjuan Zhang, Mengyi Hu, Yawei Lin, Jing He, Shuwen Li, Yulian Zhang, Hong-juan Lang

**Affiliations:** 1 Department of Nursing, Fourth Military Medical University, No.169 Changle West Road, Xi’an, 710032 Shaanxi China; 2 Department of Nursing, Shaanxi University of Chinese Medicine, Xianyang, Shaanxi China; 3 956th Hospital of the Chinese People’s Liberation Army, Tibet Xizang, China; 4 Laboratory Department, Yan’an University Affiliated Hospital, Yan’an, Shaanxi China; 5 Department of Neurosurgery, Tangdu Hospital, No.1 Xinsi Road, Xi’an, 710032 Shaanxi China; 6 Shaanxi Provincial People’s Hospital, No.256 Youyi West Road, Xi’an, 710032 Shaanxi China

**Keywords:** Nurses, Biosafety incident, Response competence, Scale, Reliability, Validity

## Abstract

**Aims:**

This study was designed to develop a biosafety incident response competence scale and evaluate its validity and reliability among clinical nurses.

**Design:**

This study employed a sequential approach, comprising four phases: (1) the establishment of a multidimensional conceptual model, (2) the preliminary selection of the items, (3) further exploration and psychometric testing of the items, (4) the application of the scale among clinical nurses.

**Methods:**

The biosafety incident response competence conceptual model was developed through literature review and the Delphi method. A total of 1,712 clinical nurses participated in the preliminary items selection, while 1,027 clinical nurses were involved in the further psychometric testing from July 2023 to August 2023. The item analysis, exploratory factor analysis and confirmatory factor analysis were conducted to evaluate the construct validity. Reliability was measured using Cronbach’s alpha, split-half reliability, and test-retest reliability, while validity analysis included content validity, structural validity, convergent validity, and discriminant validity. From September to November 2023, we conducted a survey using the established scale with a total of 4338 valid questionnaires collected. T-test and variance analysis was employed to determine potential variations in biosafety incident response competence based on participants characteristics.

**Results:**

The final scale is composed of 4 factors and 29 items, including monitoring and warning abilities, nursing disposal abilities, biosafety knowledge preparedness, and infection protection abilities. The explanatory variance of the 4 factors was 75.100%. The Cronbach’s alpha, split-half reliability and test-retest reliability were 0.974, 0.945 and 0.840 respectively. The Scale-level content validity index was 0.866. The Average Variance Extracted of the 4 factors was larger than 0.5, the Construct Reliability was larger than 0.7, and the Heterotrait-Monotrait ratio were less than 0.9. There were significant differences in the scores of response competence among nurses of different ages, working years, titles, positions, departments, marital status and participation in biosafety training (all *P* < 0.05).

**Conclusions:**

The biosafety incident response competence scale for nurses exhibits satisfactory reliability and validity, making it a valuable tool for assessing clinical nurses’ abilities in responding to biosafety incidents.

**Supplementary Information:**

The online version contains supplementary material available at 10.1186/s12912-024-01848-6.

## Introduction

 Biosafety incidents encompass a range of biosafety issues caused by human incorrect and improper activities, as well as safety concerns arising from natural biological activities [1, 2]. These incidents include but not limited to infectious disease outbreaks, animal and plant epidemic, biotechnology incidents, laboratory biosafety accidents, biological weapons and bioterrorism attacks [1, 3]. The global climate change and increased globalization have led to a heightened concern regarding the rapid spread of emerging infectious diseases [4, 5]. Globalization has also accelerated the spread of pathogenic microorganisms and increased the pathogen transmission [6]. On the other hand, biological weapons threat humanity in infecting millions of people with a deadly disease [7, 8]. Alongside these factors, the widespread application and ongoing development of biotechnology in various fields have also led to concerns regarding its misuse and abuse [9]. All of these pose a threat to the biosecurity of humanity. As a crucial aspect of national security, the biosecurity directly impacts public health, long-term stability, and sustainable development [10]. Thus, biosecurity should be considered an integral part of overall national security [11]. It is crucial to enhance the system and capacity building of epidemic prevention and control and scientific research on public health [12]. In the past few years, many drawbacks have been exposed in the response to biosafety incidents such as COVID-19 Infectious Diseases and laboratory biosafety [13, 14]. Reviews of the outbreak and handling of the COVID-19 pandemic revealed weaknesses in early monitoring and warning systems for infectious disease outbreaks [15, 16].

The biosafety incident response competence refers to the emergency preparedness, monitoring and early warning, protection and control, and disposal capabilities that individuals possess when dealing with biosafety incidents, in order to cut off the spread and transmission of biosafety infections, avoid or reduce the consequences of diseases and deaths caused by biological threats [14]. As the medical workforce, nursing staff plays a vital role in biosafety incident prevention and response [17, 18]. Nurses with good biosafety event response capabilities can efficiently treat infected patients, contain the spread of biosafety infections, and to the largest extent minimize the disease severity [18]. Their response competence is not only related to biosecurity threat warning, but also related to the effectiveness of the biosafety infection treatment [19]. Given the impact on public health and social stability, the biosafety incident response competence of nursing staff holds immense significance [20]. Therefore, it is urgent to clarify and enhance clinical nurses’ biosafety incident response competence. However, there is a lack of specialized evaluation tools assessing nursing staff’s ability in responding to biosafety events, as well as a lack of investigation into the ability of clinical nursing staff to respond to biosafety events. The existing tools evaluating the response capacity of health personnel to biosafety incidents involve the Epidemic Preparedness Index (EPI) issued by Metabiota and the Global Health Security Index (GHSI) issued by Johns Hopkins University [21, 22]. Researchers use EPI and GHSI to assess the response capacity of health professionals in handling public health emergencies caused by infectious diseases [23, 24]. And Halcomb et al. [25] have developed the Brief Coping Orientation to Problems Experienced Scale for nurses during COVID-19. While this scale addresses coping strategies in the context of infectious disease incidents, it is crucial to acknowledge that biosafety incidents encompass broader aspects beyond infectious diseases. These instruments are insufficient to measure the biosafety incident response competence for clinical nursing staff. Therefore, relying solely on EPI and GHSI may not accurately gauge the biosafety incident response competence of nursing staff and Halcomb E’s scale is not sufficient to reflect the biosafety incident response competence for clinical nurses. Thus, there is a need for a quantifiable assessment tool specifically designed to evaluate the biosafety incident response competence for clinical nursing staff.

It is urgent to develop a specialized scale for assessing the ability of nursing staff to respond to biosafety events. Such a scale would play a crucial role in clarifying the current status of nursing staff’s biosafety event response ability. It would help identify existing shortcomings, pinpoint areas requiring improvement, and serve as a valuable reference for enhancing their preparedness and response capabilities for clinical nurses in future biosafety events.

## The study

### Aims

The aim of our study was to develop a biosafety incident response competence scale specifically tailored for clinical nurses and to assess its validity and reliability among.

### Participants and sampling

#### Scale development and validation stage

We obtained permission from the administrative office of the hospital, and with their assistance, recruited clinical nurses from 7 tertiary hospitals in Shaanxi province, China by convenience sampling and the principle of voluntariness, from July 2023 to August 2023. The inclusion criteria required the clinical nurses to possess a nurse qualification certificate and engaged in clinical nursing work; Nurses who were unwilling to participate in the investigation or were not on duty during the data collection period were excluded from the study. The sample size was determined by the general rule of the factor analysis [26], which recommended an absolute sample size of at least 200 and a sample size-to-item ratio greater than 10, and a 5% sample loss rate. The first phase of the survey preliminarily explored the scale with a total of 49 items. Therefore, the sample size is N = (49 × 10) ÷ (1–5%) ≈ 516. In the second phase, which involved further exploration and evaluaion of the scale, there were a total of 33 items. Therefore, the sample size required for this phase is N = (33 × 10) ÷ (1–5%) ≈ 348. A total of 1,712 clinical nurses (for preliminary exploration of the item) and 1,027 clinical nurses (for further exploration and psychometric testing of the items) were finally recruited in our study.

#### Scale application stage

In the final phase of our study, we followed the same recruitment method as described previously. The final scale has 29 items, so the sample size required for this phase is N = (29 × 10) ÷ (1–5%) ≈ 305. From September to November 2023, we conducted a survey using the final scale and collected a total of 4338 valid questionnaires.

### Design

**Fig. 1:**
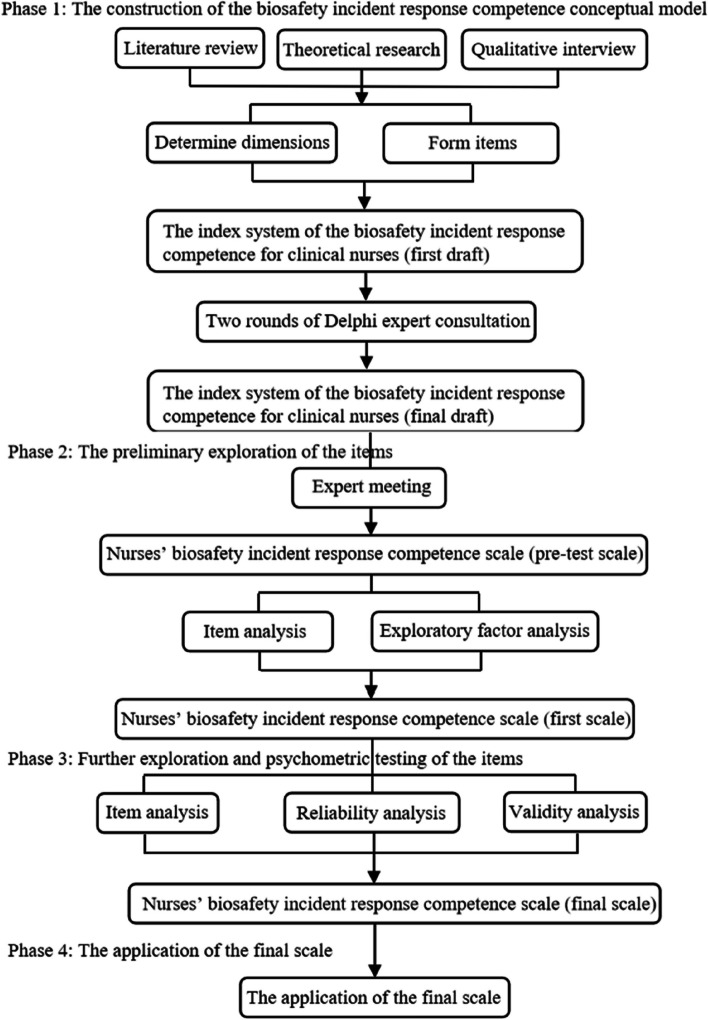
The development procedure of the biosafety incident response competence scale for clinical nurses

From July 2023 to November 2023, we conducted a mixed-method study that focused on the development, validation, and application of the Biosafety Incident Response Competence Scale for clinical nurses. It involved 4 stages: (1) the construction of the biosafety incident response competence conceptual model for clinical nurses, (2) the preliminary exploration of the biosafety incident response competence scale, (3) further exploration and psychometric testing of the biosafety incident response competence scale, (4) the application of the biosafety incident response competence scale among clinical nurses. The flowchart is shown in Fig. 1.

#### The construction of the biosafety incident response competence conceptual model for clinical nurses

In the first stage, we constructed the first draft of the biosafety incident response competence conceptual model for clinical nurses by literature review, theoretical research, one-to-one face-to-face in-depth interviews and panel discussions. The data was analyzed by Colaizzi method of phenomena observation in qualitative study [27]. The transcribed interview sessions were analyzed by the coders using thematic analysis, which was conducted in three phases: initial coding, focused coding and thematic coding [28]. According to the qualitative interviews, literature review results obtained questionnaire content item pool of 4 primary indicators (Biosafety incident preparedness, Biosafety event monitoring abilities, Biosafety infection protection competence and Biosafety incident nursing abilities), 10 secondary indicators and 48 tertiary indicators.

Due to the need for multi-party collaboration in biosafety response, we have invited experts from multiple fields: epidemiology, prevention medicine, military health service and nursing, and those who are experienced in biosafety rescue for Delphi consultation. The inclusion criteria of consultation experts were as follows: (1) a minimum of 10 years of work experience; (2) possession of an intermediate or advanced level certificate; (3) voluntary participation in the investigation. Through Delphi expert consultation, the index system was scored and modified, and the percentage level of agreement was set to 80%. After the second round of consultation, the content of the conceptual model was unanimously recognized by experts. Kendall’s concordance coefficient was used to assess the degree of agreement among the experts. In the two rounds of Delphi consultation, Kendall’s W test had statistical significance (*P*<0.01), which means the experts were in agreement. A total of 17 items were modified, 3 items were deleted, 2 items were merged, 6 items were added, and 4 items were adjusted. This led to a final draft of a 49-item pool that incorporated the valuable input and consensus achieved through the Delphi consultation.

#### Preliminary exploration of the items

In the second stage, we compiled the index system into the ‘clinical nurses’ biosafety incident response competence’ preliminary scale which included 49 items through the expert meeting. Experts transform the semantics of the conceptual model into the semantics of the scale. According to the type of questions, the Likert type responding was chosen. Once the type of Likert tool has been selected, the researcher must determine the number of Likert response options spectrum classes. In total, there is no standard guidelines for the number of response options on a Likert [29]. Since the 5-point scale form was most commonly chosen as the easiest to complete, and item omission was least frequent [30, 31], we adopted the Likert 5-level scoring method (Completely do not understand = 1, Not quite understand = 2, General = 3, Understand = 4, Very familiar = 5). Before the formal investigation, the preliminary scale was distributed to clinical nurses to ensure that the scale was easy to understand and could be used for formal investigation.

In the first round of investigation, 1,712 clinical nurses participated in the survey using the preliminary scale. Critical ratio, discrete trend, correlation coefficient, factor loading, Cronbach’s α coefficient were adopted for item analysis [32]. Exploratory factor analysis was conducted, which includes Kaiser-Meyer-Olkin analysis and factor extraction and rotation [32]. Based on the results of item analysis and exploratory factor analysis, a filtering process was undertaken to refine the items. A preliminary scale was then drafted, consisting of 4 factors (Biosafety event monitoring and warning abilities, Biosafety incident nursing disposal abilities, Biosafety knowledge preparedness and Biosafety infection protection abilities) and 33 items, basically consistent with the previous index system.

#### Further exploration and psychometric testing of the items

In the third stage, we conducted the second round of questionnaire survey which included 1,027 clinical nurses. Item analysis was performed to filter out the remaining 33 items. Additionally, exploratory factor analysis and confirmatory factor analysis were employed to re-explore and verify the structure of the scale. And we adopted reliability test and validity test to inspect the reliability and validity of the scale. Reliability analysis included measures such as test-retest reliability, Cronbach’s alpha, split-half reliability, and validity analysis included content validity, structure validity, convergent validity, discriminant validity. The sample size of 1,027 participants was randomly divided, with 514 questionnaires used for exploratory factor analysis and 513 questionnaires used for confirmatory factor analysis. Finally, the biosafety incident response competence scale with high reliability and validity was developed. The scale consisted of 4 dimensions and 29 items, offering a comprehensive assessment tool for evaluating clinical nurses’ abilities in responding to biosafety incidents.

#### The application of the final scale

In the fourth stage, we used the final scale to conduct the third round of questionnaire survey which included 4,338 clinical nurses. T-test and analysis of variance were used to compare the competence of nurses with different demographic characteristics to determine whether variations in biosafety incident response competence exist based on participant characteristics.

### Data collection

Prior to commencing the formal investigation, the researchers underwent comprehensive training on conducting the questionnaire survey. We then obtained permission from their admin office of the hospital to contact nurses by emails to distribute the electronic questionnaires. Written informed consents were obtained from the participants by electronic notification issued through email prior to conducting the study. The time for the questionnaire completion was controlled within 5 to 10 min. According to voluntary principle, we recruited 1,800 clinical nurses in the first round of investigation using convenient sampling method. A total of 1,712 valid questionnaires were collected, with a response rate of 95.11%. In the second round of investigation, 1,100 questionnaires were distributed, with 1,027 valid ones collected, leading to a response rate of 93.36%. In the third round of investigation, 4,600 questionnaires were distributed, with 4,338 valid ones collected, resulting in a response rate of 94.30%.

### Data analysis

We analyzed the data by SPSS 26.0 and Mplus 8.3. For the critical ratio, the total score of the scale was ranked from high to low, and the relationship between the top 27% and the bottom 27% was analyzed to determine the discrimination of the scale. The standard deviation of item scores represented the degree of dispersion. Items with a standard deviation below 0.85 were considered poor discriminators and recommended for removal [33]. The significant correlation coefficient indicates a strong correlation between the item and the scale. For the factor loading, if the total score is less than 0.4, the item needs to be deleted [34, 35]. And if Cronbach’s α becomes larger after deleting the item, it should be deleted [36]. For reliability analysis, we used Cronbach’s α coefficient, split-half reliability and test-retest reliability [37]. For validity analysis, we used content validity analysis, convergent validity, discriminant validity and structure analysis, which contained exploratory factor analysis and confirmatory factor analysis [38]. Content validity was assessed by the Item-level content validity index (I-CVI) and Scale-level content validity index (S-CVI). Structure validity was assessed by confirmatory factor analysis. Convergent validity was assessed by the Average Variance Extracted (AVE) and Construct Reliability (CR). Discriminant validity was assessed with the criterion of the heterotrait-monotrait ratio (HTMT). We used alpha ≤ 0.05 as the statistical difference evaluation standard.

## Results

### Characteristics of the participants

In the first round of investigation, the average age of clinical nursing staff was (32.25 ± 6.48) years old. In the second round of the survey, the average age of clinical nurses was (33.09 ± 6.36) years old. In the third round of the survey, the average age of clinical nurses was (34.03 ± 7.30) years old. Other Demographic features are shown in Table 1.

**Table 1:** General demographic data. N, number

Characteristics	The first round of investigation (*n* = 1,712)		The second round of investigation (*n* = 1,027)		The third round of investigation (*n* = 4,338)	
	N	%	N	%	N	%
Age (years)						
< 25	191	11.16	104	10.13	289	6.66
25 ~ 35	992	57.94	604	58.81	2,524	58.18
> 35	529	30.90	319	31.06	1,525	35.15
Sex						
Female	1,585	92.58	893	86.95	4,095	94.40
Male	127	7.42	134	13.05	243	5.60
Work experience in clinical nursing (years)						
< 5	457	26.69	324	31.55	689	15.88
5 ~ 10	670	39.14	421	40.99	1,469	33.86
> 10	585	34.17	282	27.46	2,180	50.25
Title						
Nurse	920	53.74	628	61.15	2,042	47.07
Nurse in charge	755	44.10	385	37.49	2,081	47.97
Deputy chief nurse or above	37	2.16	14	1.36	215	4.96
Educational background						
Below bachelor degree	116	6.78	173	16.85	475	10.95
Bachelor degree	1,574	91.94	839	81.69	3,779	87.11
Master’s degree or above	22	1.29	15	1.46	84	1.94
Department						
Department of Infectious Diseases	248	14.49	156	15.19	198	4.60
Non-infectious department	1,464	85.51	871	84.81	4,140	95.40
Positions						
Nurses	1,649	96.32	996	96.98	4,026	92.81
Head nurses	63	3.68	31	3.02	312	7.19
Marital status						
Unmarried	494	28.86	453	44.11	1,068	24.62
Married	1,200	70.09	562	54.72	3,191	73.56
Divorce or bereavement	18	1.05	12	1.17	79	1.82

### Preliminary exploration of the scale

#### Item analysis

As shown in Table 2, the results of item analysis of the 1,712 questionnaires to preliminary explore the scale in the first round showed that the values of each item in item analysis were up to the standard and all the items were to be reserved.

**Table 2:** The item analysis for clinical nurses’ biosafety incident response competence scale

Item	The first round of investigation (*n* = 1712)						Item	The second round of investigation (*n* = 1027)					
	Critical ratio	Discrete trend	Correlation coefficient	Factor loading	Cronbach’s α coefficient	Retained item		Critical ratio	Discrete trend	Correlation coefficient	Factor loading	Cronbach’s α coefficient	Retained item
1	25.115	0.925	0.611^**^	0.611	0.985	√	1	22.500	0.928	0.657^**^	0.631	0.975	√
2	27.510	0.916	0.643^**^	0.643	0.985	√	2	22.587	0.932	0.681^**^	0.677	0.975	√
3	29.896	0.926	0.686^**^	0.686	0.985	√	3	26.847	0.938	0.714^**^	0.712	0.975	√
4	25.832	0.882	0.623^**^	0.623	0.985	√	4	21.253	0.868	0.657^**^	0.645	0.975	√
5	32.128	0.876	0.730^**^	0.730	0.985	√	5	26.471	0.869	0.726^**^	0.723	0.975	√
6	34.379	0.896	0.738^**^	0.738	0.985	√	6	27.007	0.900	0.740^**^	0.730	0.975	√
7	37.160	0.947	0.757^**^	0.757	0.985	√	7	29.541	0.967	0.765^**^	0.743	0.975	√
8	33.735	0.975	0.711^**^	0.711	0.985	√	8	23.262	1.034	0.661^**^	0.672	0.975	√
9	35.179	0.969	0.754^**^	0.754	0.985	√	9	27.502	1.026	0.738^**^	0.759	0.975	√
10	39.807	0.963	0.786^**^	0.786	0.985	√	10	27.979	0.991	0.752^**^	0.718	0.975	√
11	26.732	1.051	0.624^**^	0.624	0.985	√	11	32.277	0.968	0.814^**^	0.757	0.974	√
12	31.346	1.019	0.704^**^	0.704	0.985	√	12	32.657	0.975	0.815^**^	0.775	0.974	√
13	31.358	0.977	0.736^**^	0.736	0.985	√	13	34.689	0.952	0.826^**^	0.775	0.974	√
14	27.167	0.945	0.678^**^	0.678	0.985	√	14	34.981	0.965	0.823^**^	0.747	0.974	√
15	39.318	0.935	0.819^**^	0.819	0.985	√	15	35.542	0.966	0.835^**^	0.785	0.974	√
16	40.326	0.944	0.816^**^	0.816	0.985	√	16	19.845	0.975	0.606^**^	0.553	0.975	√
17	39.994	0.923	0.829^**^	0.829	0.985	√	17	31.575	0.958	0.828^**^	0.773	0.974	√
18	39.666	0.930	0.812^**^	0.812	0.985	√	18	32.874	0.962	0.834^**^	0.780	0.974	√
19	41.176	0.950	0.823^**^	0.823	0.985	√	19	32.342	0.976	0.827^**^	0.794	0.974	√
20	28.521	0.998	0.685^**^	0.685	0.985	√	20	33.956	0.991	0.824^**^	0.740	0.974	√
21	37.172	0.928	0.823^**^	0.823	0.985	√	21	18.091	0.966	0.573^**^	0.768	0.976	×
22	39.440	0.932	0.836^**^	0.836	0.985	√	22	20.595	0.976	0.616^**^	0.796	0.975	√
23	40.588	0.943	0.840^**^	0.840	0.985	√	23	28.544	1.032	0.771^**^	0.698	0.975	√
24	39.414	0.953	0.837^**^	0.837	0.985	√	24	24.284	0.965	0.700^**^	0.688	0.975	√
25	38.919	0.978	0.827^**^	0.827	0.985	√	25	23.079	1.002	0.677^**^	0.743	0.975	√
26	33.034	0.976	0.762^**^	0.762	0.985	√	26	30.997	1.023	0.791^**^	0.713	0.975	√
27	39.152	0.950	0.838^**^	0.838	0.985	√	27	25.542	0.976	0.759^**^	0.748	0.975	√
28	23.793	1.017	0.602^**^	0.602	0.985	√	28	28.501	0.953	0.783^**^	0.776	0.975	√
29	21.771	1.003	0.581^**^	0.581	0.985	√	29	29.875	0.983	0.797^**^	0.805	0.975	√
30	37.353	1.004	0.789^**^	0.789	0.985	√	30	33.064	1.004	0.825^**^	0.808	0.975	√
31	38.911	0.966	0.829^**^	0.829	0.985	√	31	29.222	0.974	0.795^**^	0.835	0.975	√
32	28.384	0.974	0.681^**^	0.681	0.985	√	32	25.008	0.910	0.753^**^	0.781	0.975	√
33	27.842	1.004	0.679^**^	0.679	0.985	√	33	25.302	0.962	0.740^**^	0.758	0.975	√
34	35.814	1.000	0.780^**^	0.780	0.985	√							
35	38.751	1.005	0.809^**^	0.809	0.985	√							
36	42.283	0.985	0.837^**^	0.837	0.985	√							
37	39.898	0.975	0.829^**^	0.829	0.985	√							
38	42.783	0.965	0.850^**^	0.850	0.985	√							
39	38.527	0.980	0.826^**^	0.826	0.985	√							
40	41.598	0.986	0.846^**^	0.846	0.985	√							
41	39.690	0.982	0.835^**^	0.835	0.985	√							
42	40.084	0.982	0.838^**^	0.838	0.985	√							
43	34.896	0.946	0.800^**^	0.800	0.985	√							
44	35.988	0.934	0.801^**^	0.801	0.985	√							
45	36.986	0.957	0.809^**^	0.809	0.985	√							
46	39.823	0.957	0.839^**^	0.839	0.985	√							
47	35.365	0.951	0.801^**^	0.801	0.985	√							
48	33.058	0.908	0.770^**^	0.770	0.985	√							
49	32.398	0.962	0.754^**^	0.754	0.985	√							

^**^P < 0.01

#### Exploratory factor analysis

Based on the item analysis, exploratory factor analysis was employed to preliminarily identify the structure of the nursing staff’s biosafety incident response competence scale.

##### Kaiser-Meyer-Olkin (KMO) analysis

The KMO value was 0.984, the Bartley Sphericity test was statistically significant (χ^2^ = 99,415.926, *df* = 1,176, *P* < 0.001), indicating that 49 items of nursing staff’s biosafety incident response competence scale had common factors and were suitable for factor analysis.

##### Factor extraction and rotation

When extracting and rotating factors, we removed the highest factor load less than 0.4, factor load across two or more factors and the difference less than 0.2, and the number of common factors included items less than 3 [39]. According to the delete criteria, items 8, 9, 10, 14, 21, 26, 27, 31, 34, 35, 36, 37, 38, 39, 40, 41 were deleted, and 4 common factors were extracted. The cumulative contribution of variance accounted for 73.427%.

Based on the item analysis and exploratory factor analysis, a preliminary scale of nursing staff’s biosafety incident response competence was developed, which includes 4 factors and 33 items. 4 factors were named at the group meeting as follows: biosafety knowledge preparedness, biosafety event monitoring and warning abilities, biosafety infection protection abilities and biosafety incident nursing disposal abilities, as shown in Table 3.

**Table 3:** Factor matrix of clinical nurses’ biosafety incident response competence scale (*n* = 1,712)

Item	Biosafety knowledge preparedness	Biosafety event monitoring and warning abilities	Biosafety infection protection abilities	Biosafety incident nursing disposal abilities	Retained item
24	**0.715**	0.386	0.242	0.252	√
12	**0.715**	0.265	-0.006	0.306	√
23	**0.712**	0.365	0.294	0.262	√
22	**0.700**	0.372	0.312	0.246	√
17	**0.699**	0.270	0.275	0.381	√
13	**0.697**	0.281	0.066	0.342	√
11	**0.682**	0.274	-0.113	0.249	√
19	**0.671**	0.281	0.280	0.378	√
16	**0.668**	0.247	0.294	0.404	√
25	**0.652**	0.339	0.393	0.219	√
15	**0.645**	0.253	0.327	0.414	√
18	**0.631**	0.272	0.347	0.366	√
21	**0.615**	0.368	0.419	0.250	×
27	**0.609**	0.368	0.443	0.221	×
26	**0.546**	0.304	0.489	0.199	×
47	0.344	**0.788**	0.222	0.217	√
45	0.328	**0.779**	0.270	0.221	√
48	0.233	**0.757**	0.336	0.258	√
49	0.308	**0.753**	0.207	0.240	√
46	0.357	**0.738**	0.309	0.237	√
44	0.242	**0.722**	0.397	0.257	√
43	0.262	**0.698**	0.403	0.236	√
42	0.405	**0.655**	0.329	0.211	√
41	0.441	**0.608**	0.289	0.210	×
40	0.454	**0.604**	0.292	0.220	×
38	0.373	**0.559**	0.462	0.219	×
39	0.441	**0.558**	0.310	0.217	×
37	0.346	**0.514**	0.504	0.199	×
35	0.349	**0.472**	0.466	0.210	×
29	0.039	0.211	**0.824**	0.149	√
28	0.078	0.223	**0.784**	0.175	√
33	0.107	0.306	**0.779**	0.184	√
32	0.138	0.312	**0.736**	0.205	√
30	0.311	0.368	**0.652**	0.213	√
20	0.389	0.193	**0.631**	0.253	√
31	0.399	0.405	**0.588**	0.208	×
34	0.304	0.408	**0.565**	0.187	×
36	0.356	0.507	**0.519**	0.189	×
14	0.447	0.160	**0.506**	0.338	×
2	0.281	0.184	0.168	**0.715**	√
3	0.314	0.192	0.210	**0.715**	√
1	0.245	0.181	0.186	**0.698**	√
6	0.331	0.223	0.272	**0.695**	√
5	0.257	0.235	0.342	**0.695**	√
7	0.494	0.244	0.086	**0.650**	√
4	0.165	0.173	0.386	**0.638**	√
9	0.565	0.248	0.049	**0.583**	×
8	0.534	0.223	0.018	**0.569**	×
10	0.563	0.249	0.147	**0.567**	×

√: reserve item, × : delete item

### Further exploration and psychometric testing of the scale

#### Item analysis

In this section, the same method for item analysis was employed to assess the performance of each item. The results showed that item 21 met the deletion criteria and was intended to be deleted after discussion by the research group, and other items need to be reserved, as shown in Table 2.

#### Exploration of scale structure

**Fig. 2:**
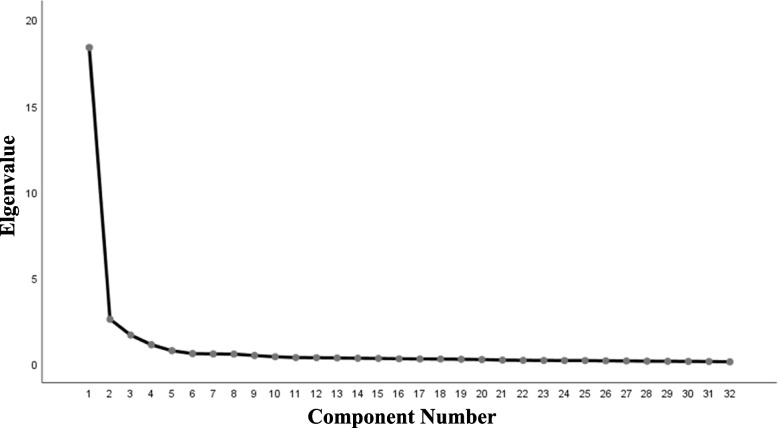
Scree plot of exploratory factor analysis for nursing staff’s biosafety incident response competence scale (*n* = 1,027)

Based on the results of the item analysis, we proceeded to conduct further exploration of the scale’s structure. The scree plot of exploratory factor analysis shows clear inflection points between components 3 ~ 5 in the scale. Based on the initial structure of the conceptual model, a 4-factor model was preliminarily extracted, as shown in Fig. 2.

We randomly selected 514 questionnaires from the 1,027 questionnaires, and exploratory factors were analyzed by principal component analysis. Bartlett sphericity test yielded a value of 16,383.411, and the KMO test value was 0.970 (*P* < 0.01). The results showed that the eigenvalues of the 4 factors were 17.925, 2.798, 1.491 and 1.128, and the variance contribution rates were 56.015%, 8.744%, 4.658% and 3.524%. The cumulative contribution of variance rate was 72.941%. However, item 9 " Regularly participate in the education of biosafety-related science knowledge “, 8 " Regularly participate in biosafety medical rescue exercises and training and joint military and civilian rescue exercises to deal with emergencies “, and 10 " Regularly pay attention to the biosafety frontier, and regularly participate in the training of biosafety nursing skills " were classified as the first common factor, falling into the same category as items 19, 18, 17, 15, 12, 11, 13, 14, and 20, given that common factor 1 corresponds to the model’s “monitoring and warning ability”. Considering that items 8, 9, and 10 do not belong to the same category as factor 1, ‘Biosafety event monitoring and warning abilities’, the research group deleted items 8–10 after discussion.

On the basis of preliminary exploratory factor analysis, the scale included 4 dimensions and 29 items. To further investigate the factor structure, another round of exploratory factor analysis was conducted using a randomly selected subset of 514 questionnaires from the pool of 1,027 valid questionnaires. The results showed that the Bartlett sphericity test value of the scale was 30,410.372, and the KMO test value was 0.973 (*P* < 0.01). The characteristic values of the four factors are 16.885, 2.370, 1.436, and 1.088, respectively, with variance contribution rates of 58.224%, 8.174%, 4.950%, and 3.751%, and cumulative variance contribution rate of 75.100%. The cumulative variance contribution rate had been improved compared to the preliminary exploratory factor analysis. Importantly, the items included in each of the four factors aligned well with the theoretical model, as depicted in Table 4. The finalized version of the scale can be found in the Supplementary file.

**Table 4:** Factor load of formal scale (29 items)

Factors and it’s items	Factor load
**Factor 1 (eigenvalue 16.885, variance contribution rate 58.224%)**	
16 Possess the ability to assess biosafety incident level, radiation impact range, severity, and medical rescue response level	0.763
15 Be able to comprehensively predict and evaluate the risk of potential complications in patients with biological infections	0.755
14 Possess the ability to assess the harm of pathogenic microorganisms	0.745
12 Understand the main points and requirements of detection and screening of pathogenic microorganisms and drug-resistant bacteria	0.740
10 Ability to identify biosafety risks	0.719
9 Monitoring of microbial resistance	0.714
8 Monitoring of common symptoms in patients with biological infections	0.697
11 Understand the quarantine points and requirements of public goods, environment, medical equipment and equipment	0.697
17 Master the reporting requirements, reporting time limit, reporting content and reporting process of different types of biosafety incidents	0.688
**Factor 2 (eigenvalue 2.370, variance contribution rate 8.174%)**	
27 Possess the ability to manage the personnel involved in biosafety emergency rescue, and be able to reasonably organize, allocate, coordinate, coordinate, guide and manage biosafety nursing work	0.788
29 Possess the ability to coordinate nursing collaboration between different departments in biosafety rescue	0.778
28 Possess the ability to communicate well with superiors and organizations to seek effective rescue assistance	0.775
25 Possess the ability to coordinate and manage biosafety medical relief materials	0.757
26 Master the key points of medical record management and record of patients with biological infection	0.720
24 Possess the ability of psychological adjust and psychological care for biologically infected patients and their families affected by infectious diseases and biological warfare agents	0.688
23 Possess a good ability to withstand pressure and psychological adjustment in the biosafety incident rescue	0.669
22 Possess the ability to properly transport and evacuate bio-infected patients	0.580
**Factor 3 (eigenvalue 1.436, variance contribution rate 4.950%)**	
3 Be familiar with biosafety incidents involving paramedics that require paramedic involvement	0.751
2Understand relevant laws and regulations such as the Biosafety Law of the People’s Republic of China, the Law of the People's Republic of China on the Prevention and Control of Infectious Diseases, and the Regulations on Biosafety Management of Pathogenic Microorganism Laboratories	0.742
5 Understand the types of pathogenic microorganisms and the transmission routes of different types of pathogenic microorganisms	0.728
1 Understand biosafety definitions, categories, hazards, and current or future potential national and international biosafety risks	0.698
6 Be familiar with the concept of antimicrobial resistance and the use of antimicrobials	0.684
4 Grasp the knowledge of care for common symptoms of patients with biological infections such as fever, chills, dizziness, headache, nausea, vomiting, diarrhea, rash, dyspnea, convulsions, and disturbance of consciousness	0.666
7 Understand the biosafety management and classification requirements of pathogenic microorganism laboratory	0.615
**Factor 4 (eigenvalue 1.088, variance contribution rate 3.751%)**	
18 Master the emergency treatment process of skin and mucous membrane exposure, respiratory mucous membrane injury, sharp instrument injury and other biosafety occupational exposure and injury	0.814
21 Be able to properly handle blood, body fluids, secretions, excreta and biosafety-related medical waste from patients with biological infections	0.773
20 Strengthen nosocomial infection control to reduce the occurrence of drug-resistant bacterial infection	0.707
13 Master the correct collection methods of blood culture samples and nasopharyngeal swabs from patients with biological infection	0.631
19 Understand the vaccination of biosafety protective vaccines	0.617

#### Reliability analysis

The scale and its 4 dimensions demonstrated good reliability. The overall internal consistency was 0.974, and that of each dimension ranged from 0.888 to 0.964. The total split-half reliability was 0.885, and of each dimension ranged from 0.856 to 0.917. To ensure comparability, we randomly selected 10% of the nursing staff and labeled them, and compared the selected nursing staff with the general nurses in Demography data, the difference was not significant (*P* > 0.05), indicating that the samples were comparable with the general samples. A questionnaire study was conducted among 10% of these nursing staff who were distributed with the scale again after a 2 weeks interval. The results showed that the total retest reliability of the scale was 0.840, and the retest reliability of each dimension was 0.696 to 0.881, as shown in Table 5.

**Table 5:** Reliability coefficient of clinical nurses’ biosafety incident response competence scale

Dimension/scale	Reliability coefficient		
	Test–retest reliability	Cronbach’s α coefficient	Split-half reliability
Biosafety event monitoring and warning abilities	0.881^**^	0.964	0.917
Biosafety incident nursing disposal abilities	0.825^**^	0.955	0.915
Biosafety knowledge preparedness	0.843^**^	0.922	0.888
Biosafety infection protection abilities	0.696^**^	0.888	0.856
Total scale	0.840^**^	0.974	0.885

^**^P < 0.01

#### Validity analysis

##### Content validity

Fifteen experts in the field of biosafety were invited to evaluate the content validity of the scale. The results showed that the Item-level content validity index (I-CVI) was 0.800 to 0.933, and Scale-level content validity index (S-CVI) was 0.866.

##### Structure validity

**Fig. 3:**
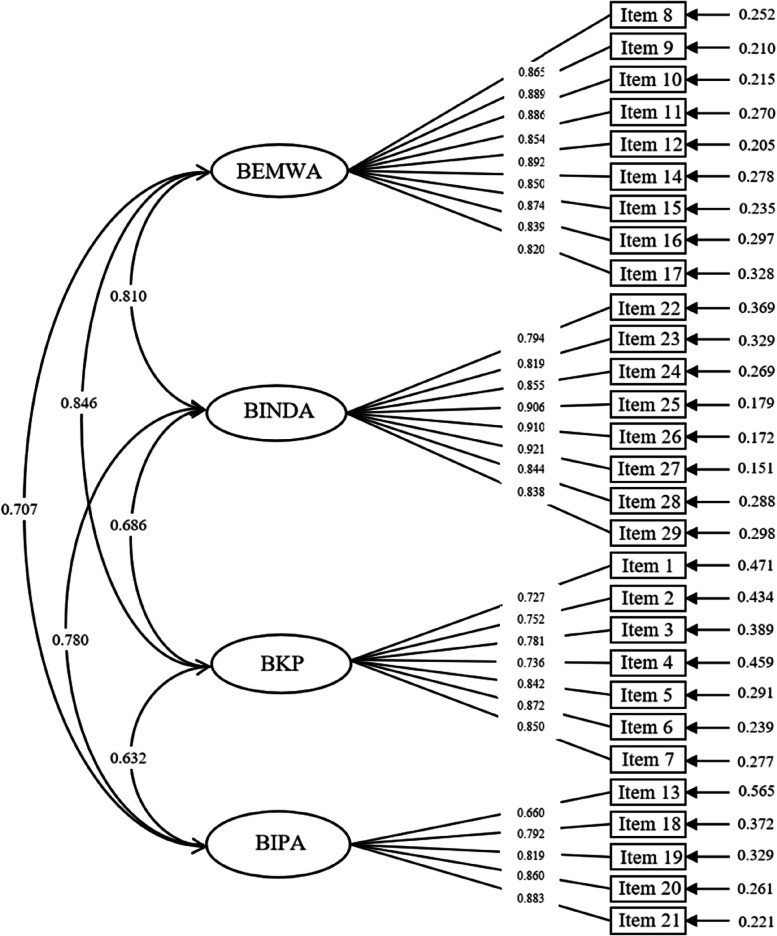
Standardized 4-factor structural model of nursing staff’s biosafety incident response competence scale (*n* = 513). BEMWA = Biosafety event monitoring and warning abilities; BINDA = Biosafety incident nursing disposal abilities; BKP = Biosafety knowledge preparedness; BIPA = Biosafety infection protection abilities

The remaining 513 questionnaires in the second round of investigation were selected for confirmatory factor analysis. The 4-factor model was fitted by the maximum likelihood estimation method. The fitting indexes were shown in Table 6, and the standard factor load model formed by confirmatory factor analysis was shown in Fig. 3. The factor load of each item was greater than 0.40, and all items had statistical significance (*P* < 0.05), indicating that the questionnaire had favorable structural validity.

**Table 6:** Confirmatory factor analysis model fitting values

Fit indexes	The measurement model	Fitting criteria
χ2/*df*	3.640	< 5.00
Root Mean Square Error Approximation (RMSEA)	0.072	< 0.08
Comparative fit index (CFI)	0.940	> 0.90
Tucker-Lewis index (TLI)	0.933	> 0.90
Standard root mean-square residual (SRMR)	0.049	< 0.80

##### Convergent validity

Convergent validity was assessed by the Average Variance Extracted (AVE) and Construct Reliability (CR). AVE above 0.5 and CR above 0.7 were considered evidence of convergent validity. AVE was larger than 0.5 for both Biosafety event monitoring and warning abilities (AVE = 0.502), Biosafety incident nursing disposal abilities (AVE = 0.524), Biosafety knowledge preparedness (AVE = 0.508) and Biosafety infection protection abilities (AVE = 0.515) attesting the convergent validity of all the first order constructs [40, 41] (See Table 7).

**Table 7:** Convergent validity evidence of the scale

Dimension	Item	Standardized factor load	AVE	CR
Biosafety event monitoring and warning abilities	Item 8	0.743	0.502	0.900
	Item 9	0.727		
	Item 10	0.721		
	Item 11	0.723		
	Item 12	0.707		
	Item 14	0.690		
	Item 15	0.691		
	Item 16	0.694		
	Item 17	0.680		
Biosafety incident nursing disposal abilities	Item 22	0.787	0.524	0.897
	Item 23	0.779		
	Item 24	0.773		
	Item 25	0.755		
	Item 26	0.716		
	Item 27	0.716		
	Item 28	0.676		
	Item 29	0.565		
Biosafety knowledge preparedness	Item 1	0.744	0.508	0.878
	Item 2	0.723		
	Item 3	0.762		
	Item 4	0.719		
	Item 5	0.718		
	Item 6	0.686		
	Item 7	0.627		
Biosafety infection protection abilities	Item 13	0.816	0.515	0.840
	Item 18	0.776		
	Item 19	0.736		
	Item 20	0.593		
	Item 21	0.642		

##### Discriminant validity

Evidence of discriminant validity between first order constructs was assessed with the criterion of the heterotrait-monotrait ratio (HTMT). HTMT below 0.9 was considered evidence of discriminant validity [42]. According to the HTMT, more liberal criterion discriminant validity was observed between the 4 engagement constructs (See Table 8).

**Table 8:** Heterotrait-Monotrait ratio of discriminant validity evidence

Heterotrait-Monotrait ratio (HTMT)	1	2	3	4
1. Biosafety event monitoring and warning abilities	-			
2. Biosafety incident nursing disposal abilities	0.817	-		
3. Biosafety knowledge preparedness	0.824	0.689	-	
4. Biosafety infection protection abilities	0.733	0.806	0.676	-

### The application of the scale

T-test and analysis of variance showed significant differences in the scores of response competence among clinical nurses of different ages, working years, titles, positions, department, marital status and participation in biosafety related training (all *P* < 0.05) (See Table 9).

**Table 9:** Comparison of biosafety incident response competence of clinical nurses with different demographic characteristics (*N* = 4,338)

Demographic characteristics	Biosafety incident response competence
Age (years)	
< 25	95.53 ± 19.96^ab^
25 ~ 35	90.92 ± 21.41
> 35	90.70 ± 22.55
*t/F*	6.348
*P*	0.02
Work years	
< 5	94.53 ± 20.79^ab^
5 ~ 10	91.35 ± 21.46
> 10	89.95 ± 22.14
*t/F*	11.778
*P*	< 0.01
Sex	
Female	91.09 ± 21.67
Male	92.12 ± 23.11
*t/F*	-0.714
*P*	0.475
Title	
Nurse	92.16 ± 21.88^a^
Nurse in charge	89.8 ± 21.62^c^
Deputy chief nurse or above	94.59 ± 21.08
*t/F*	8.937
*P*	< 0.01
Educational background	
Below bachelor degree	90.68 ± 23.03
Bachelor degree	91.23 ± 21.67
Master's degree or above	90.49 ± 18.05
*t/F*	0.174
*P*	0.841
Department	
Department of Infectious Diseases	97.96 ± 21.98
Non-infectious department	90.83 ± 21.69
*t/F*	4.518
*P*	< 0.001
Positions	
Nurses	90.81 ± 21.76
Head nurses	95.59 ± 21.23
*t/F*	-3.744
*P*	< 0.01
Marital status	
Unmarried	93.87 ± 22.12^ab^
Married	90.34 ± 21.45
Divorce or bereavement	87.04 ± 25.68
*t/F*	11.982
*P*	< 0.01
Whether participated in biosafety related training	
No	87.24 ± 20.88
Yes	98.35 ± 21.50
*t/F*	-16.55
*P*	< 0.01

^a^Comparison of the first and second items (*P* < 0.05)

^b^Comparison of the first and third items (*P* < 0.05)

^c^Comparison of the second and third items (*P* < 0.05)

## Discussion

The biosafety incident response competence scale for clinical nurses was developed using a rigorous scientific approach. A conceptual model was created based on literature review, qualitative interviews, group meetings, and Delphi consultation. The research group used the Classic Test Theory (CTT) to assess item quality, including measures such as critical ratio, discrete trend, the correlation coefficient, factor loading and Cronbach’s α coefficient [43, 44]. On the basis of the conceptual model, the structure of the scale was examined, and its reliability and validity were tested. The final version of scale consisted of 4 dimensions and 29 items, demonstrating favorable reliability and validity. The cumulative variance contribution rate of the 4 factors in the scale amounted to 75.100%, indicating that these 4 factors could adequately explain the variation in biosafety incident response competence of clinical nurses to the extent of 75.100%. Reliability analysis refers to the consistency, stability, and reliability of test results [45]. Validity analysis refers to the validity of the results, that is, the consistency between the measurement results and the content to be examined [46]. The reliability and the validity test of the scale shows that it has good reliability and validity, which also reflects the scientific process of scale development. The results of research on the application of the scale showed that there were significant differences in the scores of response competence among nurses of different ages, working years, titles, positions, department, marital status and whether participated in biosafety related training, which further validated the effectiveness of the scale.

The scale developed in our study measures the biosafety incident response competence of nursing staff across four dimensions: biosafety event monitoring and warning abilities, biosafety incident nursing disposal abilities, biosafety knowledge preparedness, and biosafety infection protection abilities. The scale adopts Likert’s 5-level scoring method, where higher scores reflect a greater level of biosafety incident response competence among nursing staff. The scale developed in our study fills the existing gap in assessing the ability of nursing staff to respond to biosafety incidents by providing a much-needed measurement tool.

Currently, the world is facing various biosecurity threats, including emerging infectious diseases, bioterrorism attacks, and the potential use of biological weapons [47]. The recurrence of public health emergencies and safety incidents such as COVID-19 and other emerging infectious diseases underscored the significance of biosafety [48]. Meanwhile, the escalating safety risks associated with the misuse and abuse of new dual-use biotechnology have drawn unprecedented global attention to biosafety issues[49]. Nursing staff are at the forefront of clinical care, directly engaging with patients. They are not only “sentries” who discover dangerous situations, but also the first “shield” to deal with threats [50]. Therefore, quantifying their ability to respond to biosafety incidents is crucial [51, 52].

Biosafety event monitoring and warning abilities dimension includes 9 items. Biosafety incidents are hidden and uncertain, which will lead to serious consequences if not identified timely [53]. Therefore, clinical nurses need to possess strong monitoring and warning capabilities to identify biosafety risks. Biosafety incident nursing disposal abilities dimension includes 8 items. In the event of a biosafety incident, nursing staff with proficient nursing disposal abilities can swiftly carry out rescue operations, contributing to disease containment and promoting patient recovery [54, 55]. Research showed that good nursing disposal ability could achieve timely treatment of patients [56]. Biosafety knowledge preparedness dimension includes 7 items. Currently, biosafety has emerged as a critical aspect of national security, and everyone needs to basically acquire biosafety knowledge [2, 57]. Existing research shows that caregivers lack preparedness when dealing with emergencies and disasters [58]. Therefore, it is essential for clinical nurses, particularly those specializing in biosafety nursing, to acquire comprehensive knowledge of biosafety. As crucial participants in managing biosafety incidents, nursing staff should possess systematic knowledge of biosafety to effectively respond to such threats [59]. Biosafety infection protection abilities dimension includes 5 items. A study of SARS-CoV-2 infection among healthcare workers in Colombia shown that medical personnel are susceptible to infections while responding to infectious diseases [60]. Due to the fact that most biosafety incidents can be contagious, nursing staff also need to prioritize self-protection during rescue operations and maintain a strong preventive mindset [61]. The biosafety guidance is intended to provide insights to nurses regarding the proper methods of handling the blood and other body fluid samples for biochemical investigations concerning the proper methods of sample collection, transport, processing, and disposal [50].

The dimensions of the scale and the abilities in the items cover each stage of nursing staff’s biosafety event rescue, providing detailed content to effectively assess their competence in responding to biosafety events. This scale can serve as a valuable tool for assessing, evaluating, and training nursing staff in biosafety event response. In future research, we can use this scale to measure the biosafety incident response competence of clinical nurses, identify their weaknesses, and carry out targeted training to improve their biosafety incident response competence.

Our research also has certain limitations. In our study, we didn’t conduct cross-group measurement invariance analysis on nurses from different levels of hospitals and departments. So, it remains unclear whether there were differences in its application among different groups with distinct characteristics.

## Conclusion

The present study explained the steps we took to develop a new tool to measure the biosafety incident response competence for clinical nurses. The final scale is composed of 4 factors and 29 items, including monitoring and warning abilities, nursing disposal abilities, biosafety knowledge preparedness, and infection protection abilities. The exploration method, psychometric testing, reliabilition and validition approaches we used (item analysis, exploratory factor analysis, Cronbach’s alpha, split-half reliability, test-retest reliability, content validity, structure validity, convergent validity, discriminant validity) provided justification for the satisfactory reliability and validity.

### Supplementary Information

**Supplementary Material 1.**

## Data Availability

The datasets generated and analyzed during the current study are not publicly available due to the protection of the privacy of clinical nurses but are available from the corresponding author (906963251@qq.com) on reasonable request.
