# Supplementary material for: The biosafety incident response competence scale for clinical nursing staff: a development and validation study
Source: BMC Nurs. 2024 Mar 14;23:180. doi: 10.1186/s12912-024-01848-6 (PMC10941487; doi:10.1186/s12912-024-01848-6)
Supplement: Supplementary file 1 — Supplementary Material 1. [file 12912_2024_1848_MOESM1_ESM.docx]

**The biosafety incident response competence scale for clinical nursing staff**

1.Possess the ability to assess biosafety incident level, radiation impact range, severity, and medical rescue response level.

A Completely do not understand; B Not quite understand; C General; D Understand; E Very familiar.

2.Be able to comprehensively predict and evaluate the risk of potential complications in patients with biological infections.

A Completely do not understand; B Not quite understand; C General; D Understand; E Very familiar.

3.Possess the ability to assess the harm of pathogenic microorganisms.

A Completely do not understand; B Not quite understand; C General; D Understand; E Very familiar.

4.Understand the main points and requirements of detection and screening of pathogenic microorganisms and drug-resistant bacteria.

A Completely do not understand; B Not quite understand; C General; D Understand; E Very familiar.

5.Ability to identify biosafety risks.

A Completely do not understand; B Not quite understand; C General; D Understand; E Very familiar.

6.Monitoring of microbial resistance.

A Completely do not understand; B Not quite understand; C General; D Understand; E Very familiar.

7.Monitoring of common symptoms in patients with biological infections.

A Completely do not understand; B Not quite understand; C General; D Understand; E Very familiar.

8.Understand the quarantine points and requirements of public goods, environment, medical equipment and equipment.

A Completely do not understand; B Not quite understand; C General; D Understand; E Very familiar.

9.Master the reporting requirements, reporting time limit, reporting content and reporting process of different types of biosafety incidents.

A Completely do not understand; B Not quite understand; C General; D Understand; E Very familiar.

10.Possess the ability to manage the personnel involved in biosafety emergency rescue, and be able to reasonably organize, allocate, coordinate, coordinate, guide and manage biosafety nursing work.

A Completely do not understand; B Not quite understand; C General; D Understand; E Very familiar.

11.Possess the ability to coordinate nursing collaboration between different departments in biosafety rescue.

A Completely do not understand; B Not quite understand; C General; D Understand; E Very familiar.

12.Possess the ability to communicate well with superiors and organizations to seek effective rescue assistance.

A Completely do not understand; B Not quite understand; C General; D Understand; E Very familiar.

13.Possess the ability to coordinate and manage biosafety medical relief materials.

A Completely do not understand; B Not quite understand; C General; D Understand; E Very familiar.

14.Master the key points of medical record management and record of patients with biological infection.

A Completely do not understand; B Not quite understand; C General; D Understand; E Very familiar.

15.Possess the ability of psychological adjust and psychological care for biologically infected patients and their families affected by infectious diseases and biological warfare agents.

A Completely do not understand; B Not quite understand; C General; D Understand; E Very familiar.

16.Possess a good ability to withstand pressure and psychological adjustment in the biosafety incident rescue.

A Completely do not understand; B Not quite understand; C General; D Understand; E Very familiar.

17.Possess the ability to properly transport and evacuate bio-infected patients.

A Completely do not understand; B Not quite understand; C General; D Understand; E Very familiar.

18.Be familiar with biosafety incidents involving paramedics that require paramedic involvement.

A Completely do not understand; B Not quite understand; C General; D Understand; E Very familiar.

19.Understand relevant laws and regulations such as the Biosafety Law of the People's Republic of China, the Law of the People's Republic of China on the Prevention and Control of Infectious Diseases, and the Regulations on Biosafety Management of Pathogenic Microorganism Laboratories.

A Completely do not understand; B Not quite understand; C General; D Understand; E Very familiar.

20.Understand the types of pathogenic microorganisms and the transmission routes of different types of pathogenic microorganisms.

A Completely do not understand; B Not quite understand; C General; D Understand; E Very familiar.

21.Understand biosafety definitions, categories, hazards, and current or future potential national and international biosafety risks.

A Completely do not understand; B Not quite understand; C General; D Understand; E Very familiar.

22.Be familiar with the concept of antimicrobial resistance and the use of antimicrobials.

A Completely do not understand; B Not quite understand; C General; D Understand; E Very familiar.

23.Grasp the knowledge of care for common symptoms of patients with biological infections such as fever, chills, dizziness, headache, nausea, vomiting, diarrhea, rash, dyspnea, convulsions, and disturbance of consciousness.

A Completely do not understand; B Not quite understand; C General; D Understand; E Very familiar.

24.Understand the biosafety management and classification requirements of pathogenic microorganism laboratory.

A Completely do not understand; B Not quite understand; C General; D Understand; E Very familiar.

25.Master the emergency treatment process of skin and mucous membrane exposure, respiratory mucous membrane injury, sharp instrument injury and other biosafety occupational exposure and injury.

A Completely do not understand; B Not quite understand; C General; D Understand; E Very familiar.

26.Be able to properly handle blood, body fluids, secretions, excreta and biosafety-related medical waste from patients with biological infections.

A Completely do not understand; B Not quite understand; C General; D Understand; E Very familiar.

27.Strengthen nosocomial infection control to reduce the occurrence of drug-resistant bacterial infection.

A Completely do not understand; B Not quite understand; C General; D Understand; E Very familiar.

28.Master the correct collection methods of blood culture samples and nasopharyngeal swabs from patients with biological infection.

A Completely do not understand; B Not quite understand; C General; D Understand; E Very familiar.

29.Understand the vaccination of biosafety protective vaccines.

A Completely do not understand; B Not quite understand; C General; D Understand; E Very familiar.
